# A meta-analysis of the value of MRI-based VBQ scores for evaluating osteoporosis

**DOI:** 10.1016/j.bonr.2023.101711

**Published:** 2023-08-26

**Authors:** Ang Chen, Shangyong Feng, Lijuan Lai, Caifeng Yan

**Affiliations:** Department of Endocrinology, Northern Jiangsu People's Hospital, The First Clinical College of Dalian Medical University, Yangzhou 225001, Jiangsu, China

**Keywords:** MRI, VBQ, Osteoporosis, Meta-analysis

## Abstract

**Objective:**

Osteoporosis is the most common skeletal disease in humans. Early onset of osteoporosis is usually asymptomatic, so early diagnosis is critical. The purpose of this study was to analyze the value of MRI-based VBQ scores for evaluating osteoporosis.

**Methods:**

We searched PubMed, Embase, the Cochrane Library databases, Web of Science, and some Chinese electronic databases for published articles and the ClinicalTrials.gov site for completed but unpublished studies on evaluating the value of MRI-based VBQ scores for evaluating osteoporosis. We calculated the summarized sensitivity, specificity, the ROC curve (AUC) values and their 95% confidence intervals (CIs) using MetaDiSc 1.4 software and STATA.

**Results:**

Our study included 8 studies involving 999 patients of which 660 patients were diagnosed with osteopenia/osteoporosis, and 339 patients were identified as having normal BMD. The pooled sensitivity was 0.809 (95% CI, 0.777–0.838, $I^{2}$ = 78.8%), the pooled specificity was 0.640 (95% CI, 0.587–0.691, $I^{2}$ = 85.9%), and the pooled AUC was 0.8375.

**Conclusion:**

MRI-based VBQ scores provided high sensitivity and moderate specificity in detecting osteoporosis. Opportunistic use of VBQ scores could be considered, e.g. before lumbar spine surgery.

**Prospero registration number:**

CRD42022377024.

## Highlights

• Aim of this study is the value of MRI-based VBQ scores for evaluating osteoporosis.
• The validity of using MR images to estimate osteoporotic status.
• Opportunistic use of VBQ scores could be considered to screen osteoporosis.

## 1 Introduction

Osteoporosis is the most common skeletal disease in humans, characterized by reduced bone density, and is increasingly common in postmenopausal women and the general population over the age of 50 (Cosman et al., 2014a). Nearly 200 million people worldwide are diagnosed with osteoporosis each year, and nearly 9 million osteoporotic fractures occur each year (Yaacobi et al., 2017). Osteoporotic fractures occur mostly in older patients, which exhibit underlying and unfavorable systemic diseases (Smith et al., 1975). Because of the complexity of treatment and poor prognosis, osteoporotic fractures have the highest hospitalization costs, followed by myocardial infarction and stroke (Singer et al., 2015). Early onset of osteoporosis is usually asymptomatic, so early diagnosis is critical for osteoporosis (Camacho et al., 2020).

According to the guidelines, the currently common method for diagnosing osteoporosis is dual-energy X-ray absorptiometry (DXA) scanning (Cosman et al., 2014b). However, DXA scans from the hip or spine alone are not accurate enough to diagnose osteoporosis. Spinal deformities, previous compression fractures, and aortic atherosclerosis can lead to an increase in effective X-ray uptake, resulting in a pseudo-elevation of the T-Score (Rumancik et al., 2005; Girardi et al., 2001). It has been shown that measuring vertebral signal intensity on T1-weighted images of lumbar spine MRI can be used to evaluate bone quality, and it is more sensitive than DXA (Xie et al., 2019). Some studies have shown that the development of osteoporosis was often accompanied by an increase in bone marrow adipocytes and changes in the content of various bone marrow adipose tissues (Meunier et al., 1971; Shah and Hanrahan, 2011a), and bone marrow signal intensity was negatively correlated with bone mineral density (BMD) (Shen et al., 2012). Vertebral body bone quality (VBQ) was a new method for estimating bone density, which was based on T1-weighted images of the lumbar spine, and it was probably useful in evaluating vertebral bone quality and osteoporosis by measuring the degree of fat infiltration within the vertebral body (Ehresman et al., 2020a).

Many studies have reported that VBQ scores had a correlation with the DXA T-scores and it could evaluate bone quality. VBQ scores are based on non-contrast, T1-weighted MRIs of the lumbar spine, which are calculated by first placing regions of interest (ROI) within the medullary portions of the L1-L4 vertebral bodies and within the cerebrospinal fluid space at the level of L3, and average signal intensities (SIs) within each ROI were recorded along with the average SI of the CSF. The VBQ was then measured as the quotient of the median SI of the vertebrae divided by the SI of the CSF (Eq. (1)) (Ehresman et al., 2020a). Ehresman et al. found that VBQ significantly differentiated between healthy and osteopenic/osteoporotic bone and it correlated moderately with the overall minimum T-scores of the femoral neck (Ehresman et al., 2021). VBQ score was negatively correlated with T-score, with high VBQ score representing low bone mass. Kadri et al. believed that the VBQ is a simple and effective tool that may help identify which patients have skeletal diseases (Kadri et al., 2022). Salzmann et al. concluded that the VBQ score had demonstrated moderate diagnostic power in distinguishing between patients with normal BMD and those with osteopenic/osteoporotic BMD, and it may be an opportunistic imaging for estimating bone density in the future (Salzmann et al., 2022). Recently, many studies had been published on the use of VBQ scores to evaluate osteoporosis. Therefore, we performed this meta-analysis to explore the value of MRI-based VBQ scores for evaluating osteoporosis.

(1) $$\mathrm{VBQ}\;\text{score}=\frac{{\mathrm{SI}}_{L1-L4}}{{\mathrm{SI}}_{\mathrm{CSF}}}$$

## 2 Materials and methods

### 2.1 Data sources and search strategy

We performed a systematic search of the following electronic databases: PubMed, Embase, the Cochrane Library databases, and Web of Science, and searched some Chinese electronic databases. We also searched the ClinicalTrials.gov site for completed but unpublished studies. The search included articles published from inception to November 4, 2022. Our search strategy was based on the following keywords: (Osteoporosis OR Bone Loss OR osteopenia) AND (magnetic resonance imaging OR MRI OR MR) AND VBQ. The full details of our search were registered on the PROSPERO database (CRD42022377024).

### 2.2 Study selection

The articles met the following conditions in this study: the study population was adults >18 years old; MRI and DXA of the lumbar spine were performed, and the interval between the two examinations was less than six months; it reported suffificient data to construct 2 × 2 contingency tables with at least ten patients. The exclusion criteria: patients with a history of internal fixation devices and surgery in the lumbar spine; patients with congenital spinal deformities, spinal tumors, spinal infections, ankylosing spondylitis and other forms of arthritis.

### 2.3 Data extraction and quality assessment

Two investigators independently extracted the following information from the included studies and quality assessment of studies: the first author, the year of publication, the characteristics of the study, the sample size, the characteristics of the study population, the specifications regarding the methodology, the numbers of true/false positives, and true/false negatives. Study quality was assessed according to the Quality Assessment of Diagnostic Accuracy Studies (QUADAS-2) tool to determine the presence of bias and the clinical applicability in the included studies (Whiting et al., 2011). Another investigator addressed any conflicts by discussion and consensus.

### 2.4 Study quality

The QUADAS-2 tool consists of 4 main domains: patient selection, index text, reference standard, flow and timing. All 4 domains are evaluated for risk of bias. For applicability concerns, only the first 3 domains are evaluated. Risk of bias is judged as “low,” “high,” or “unclear.” If the answers to all signaling questions for a domain are “yes,” then risk of bias can be judged low. If any signaling question is answered “no,” potential for bias exists. The “unclear” category should be used only when insufficient data are reported to permit a judgment (Leeflang et al., 2013).

### 2.5 Statistical analysis

Statistical data analysis were performed using MetaDiSc 1.4 software and STATA (version 14.0, STATA Corp., Texas USA) with the MIDAS module (Zamora et al., 2006). Diagnostic accuracy data (true/ false positive and true/ false negative) extracted from these studies were combined to calculate sensitivity, specificity, positive likelihood ratio (PLR), negative likelihood ratio (NLR), diagnostic ratio (DOR), and area under the ROC curve (AUC) values for all individual studies and their corresponding pooled measurements at 95% CI. The AUC is often used as a summary measure of the receiver operating characteristic (sROC) curve. It represents the overall performance of a diagnostic test at various diagnostic thresholds and is used to differentiate between the accuracy of cases and non-cases of a disease. The sROC curve describes the relationship between test sensitivity and specificity across studies (Walter, 2005). Heterogeneity between studies was caused by threshold and non-threshold effects. The presence of heterogeneity due to threshold effects was explored by calculating the Spearman correlation coefficient between the log of sensitivity and the log of (1-specificity) and by plotting the sROC curve; heterogeneity due to non-threshold effects was explored by the Cochran-Q test of the DOR and the I^2^ of the evaluation index of the diagnostic test described above. Influence analysis and publication bias test on the data of this study were performed by using STATA 14.0.

## 3 Results

### 3.1 Study selection

**Fig. 1:**
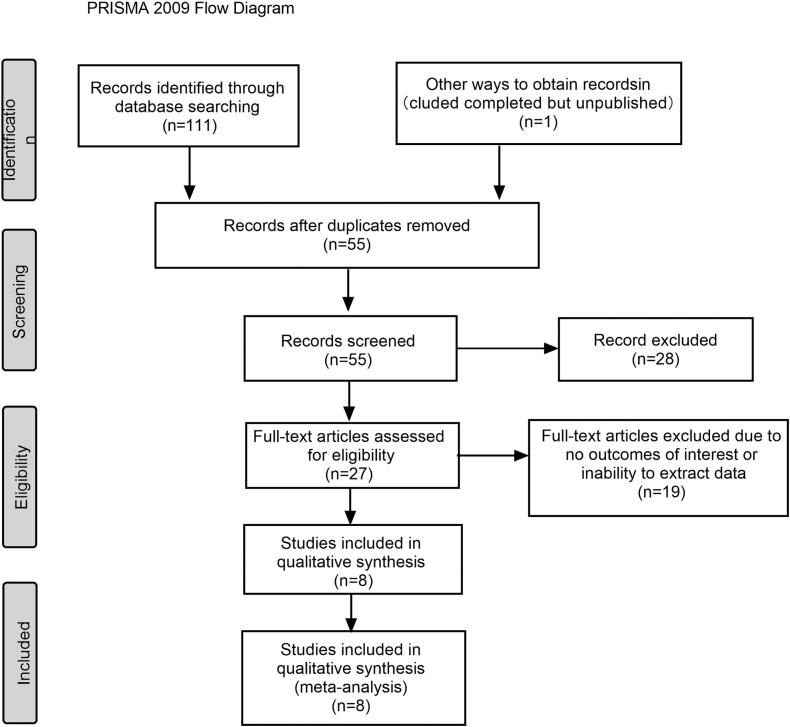
Flow diagram of study selection for analysis A PRISMA flow diagram demonstrating the study selection after screening and full text review.

The whole study selection process was represented in the PRISMA 2009 flow diagram (Fig. 1). A total of 112 articles were retrieved through the search of electronic databases. After removed duplicate articles, the titles and abstracts of the remaining 55 articles were read, 28 articles were not consistent with our inclusion criteria, and then 27 articles were read in full text. 21 studies were excluded because they either did not report results of interest or from which 2×2 columnar data could not be extracted. Finally, 8 studies (Kadri et al., 2022; Salzmann et al., 2022; Hao et al., 2023; Chang et al., 2021; Kim et al., 2023; Haffer et al., 2022; Huang et al., 2022; 王平川, 王俊武, 石鹏志, 等, 2022) that involved 999 patients were included in this study.

### 3.2 Study and patients' characteristics

Table 1 shows the detailed characteristics of the patients that were included in this study. 8 studies were included in the meta-analysis, all included studies were retrospective and included a total of 999 patients. According to the WHO diagnostic criteria (Kanis et al., 2000), the patients were classified according to T-score as healthy (T-score ≥ −1), osteopenia (−2.5 < T-score < −1), and osteoporosis (T-score ≤ −2.5 or a history of a hip or spine fracture), of which 660 patients were diagnosed with osteopenia/osteoporosis, and 339 patients were identified as having normal BMD. The average age ranged from 56 to 70 years old, the average BMI ranged from 23.6 Kg/m2 to 31.5 Kg/m2, the minimum T-score ranged from −2.53 to −0.39, and the average VBQ score ranged from 2.20 to 3.80.

**Table 1:** Characteristics of 8 studies included in the meta-analysis.

First author	Year of publication	Ref. no.	Duration of recruitment	No. of patients with osteopenia/osteoporosis	No. of patients with normal bone mass	Threshold for VBQ		Age (year,mean ± SD/mean)	Sex (male) [Number (%)]	BMI (Kg/㎡,mean ± SD/mean)	DXA T-score (mean ± SD/mean)			VBQ scores (mean ± SD/mean)	MRI Sequence	Magnetic strength
											Femoral neck T-score	Hip T-score	Minimum T-score			
Liu Hao	2022	(Hao et al., 2023)	01/2017–06/2021	115	48	3.08	Osteopenia/osteoporosis group	68.6±6.3	0(0)	24.8±3.9	−2.20±0.71	−1.96±0.79	−2.53±0.65	3.42±0.38	T1-weighted MR image	1.5 T
							Normal bone mass group	66.9±6.3	0(0)	25.5±3.6	−0.36±0.57	−0.36±0.47	−0.62±0.34	2.86±0.47		
Hsuan-Kan Chang	2022	(Chang et al., 2021)	2020–2021	32	30	NR		61.9±12.9	20(32.3)	24.4±3.3	−0.95±1.72 (T-score of lumbar spine)			3.42±0.75	T1-weighted MR image	3.0 T
A. Kadri	2022	(Kadri et al., 2022)	01/09/2017–01/09/2020	66	17	3.01		70.1±8.4	17(20.5)	28.9±6.0	−1.52±0.91	NR	−1.97±1.07	3.39±0.68	T1-weighted MR image	1.5/3.0 T
Ashley Yeo Eun Kim	2022	(Kim et al., 2023)	01/2016–05/2021	40	21	2.60		64.0±12.0	24(39.3)	28.1±5.9	NR	NR	−1.60±1.60	2.40±0.60	T1-weighted MR image	NR
Henryk Haffer	2022	(Haffer et al., 2022)	2014–2021	174	93	2.18	Osteopenia/osteoporosis group	67.1	75(43.1)	29.6	NR	NR	NR	3.04	T1-weighted MR image	NR
							Normal bone mass group	57.8	43(46.2)	30.2	NR	NR	NR	2.57		
Weibo, Huang	2022	(Huang et al., 2022)	09/2020–03/2022	63	20	2.91	Osteopenia/osteoporosis group	67.7±7.1	26(41.3)	23.6±3.0	−1.95±0.66	−1.49±0.83	−2.04±0.70	3.80±0.81	T1-weighted MR image	1.5/3.0 T
							Normal bone mass group	66.1±7.2	11(55.0)	24.3±2.4	−0.27±0.69	−0.31±0.71	−0.39±0.68	2.99±0.79		
Stephan N. Salzmann	2022	(Salzmann et al., 2022)	2014–2019	128	70	2.39	Osteopenia/osteoporosis group	64.9±9.1	58(45.3)	27.7±5.6	NR	NR	NR	2.60±0.60	T1-weighted MR image	NR
							Normal bone mass group	56.7±12.4	36(51.4)	29.0±6.0	NR	NR	NR	2.20±0.50		
Pingchuan, Wang	2022	(王平川, 王俊武, 石鹏志, 等, 2022)	01/2019–08/2020	42	40	2.98	Osteopenia/osteoporosis group	70.1±7.7	10(23.8)	28.4±4.9	−2.10	−1.90	−2.20	3.22±0.48	T1-weighted MR image	1.5 T
							Normal bone mass group	63.1±9.8	15(37.5)	31.5±5.4	−0.60	−0.5	−0.70	2.30±0.37		

Abbreviation: NR not reported, T tesla.

### 3.3 Assessment of study quality

**Fig. 2:**
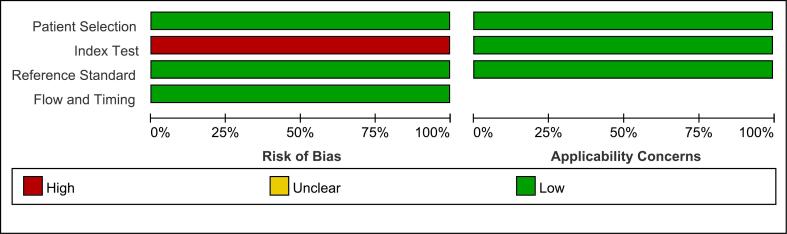
Risk of bias and applicability concerns graph: review authors' judgements about each domain presented as percentages across included studies.

**Fig. 3:**
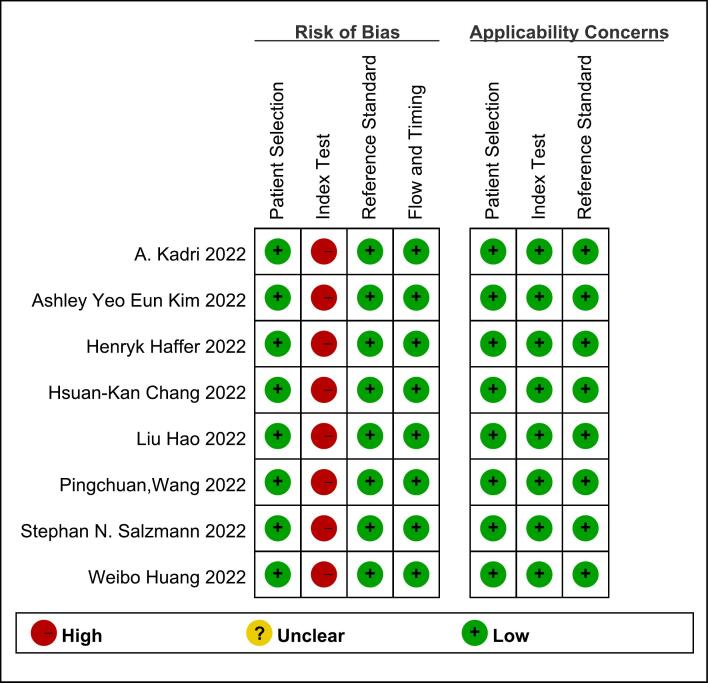
Risk of bias and applicability concerns summary: review authors' judgements about each domain for each included study.

In the risk of bias evaluation section, the patient selection domain assessed whether the selection of cases were biased: all trials included in this study were consecutive; all case-control studies were avoided; and there were no unjustified exclusions of cases, which was a low risk of bias. The “index test” was a high risk of bias, this domain was to assess whether the implementation or interpretation of the index test was subject to bias: MRI interpretation in the included studies was performed without knowledge of the results of DXA; however, none of the included studies had a predefined threshold, because the MRI-based VBQ scores were less likely to assess bone quality and did not have a defined threshold. The reference standard domain assessed whether the implementation and interpretation of the reference standard was subject to bias: the reference standard correctly distinguished between disease states (DXA clarified the diagnosis of osteoporosis); the interpretation of the results of the reference standard (DXA) is fully blinded, so this domain was a low risk of bias. The flow and timing domain assessed whether there was bias in the flow of cases: there was a reasonable time interval between included studies (no >6 months between DXA and MRI); all cases were subjected to the same and only reference standard (DXA); and there were no missed cases, so the domain was also a low risk of bias. In the applicability concerns evaluation section, the risks were low of “patient selection,” “index test,” and “reference standard”. The patient selection domain assessed whether the included patients and context matched the evaluation questions: included patients matched the evaluation value of MRI-based VBQ scores for evaluating osteoporosis. The Index test domain evaluated whether the implementation and interpretation of the index test matched the evaluation question: the VBQ scores were applicable to the evaluation of osteoporosis. The reference standard domain evaluated the applicability of the reference standard: DXA was applicable to the evaluation of osteoporosis. In summary, we considered the quality of included articles to be high. Fig. 2, Fig. 3 showed graphical representations of the QUADAS-2 risk assessment.

### 3.4 Diagnostic accuracy of VBQ scores for evaluating osteoporosis

**Fig. 4:**
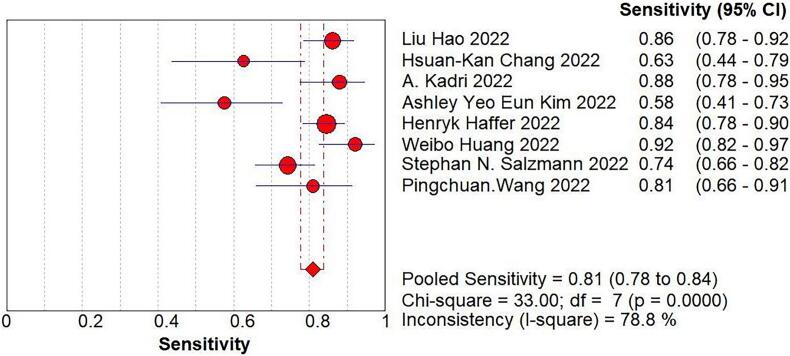

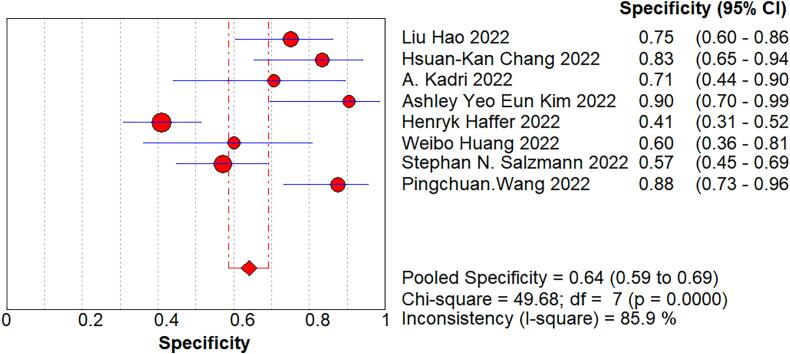

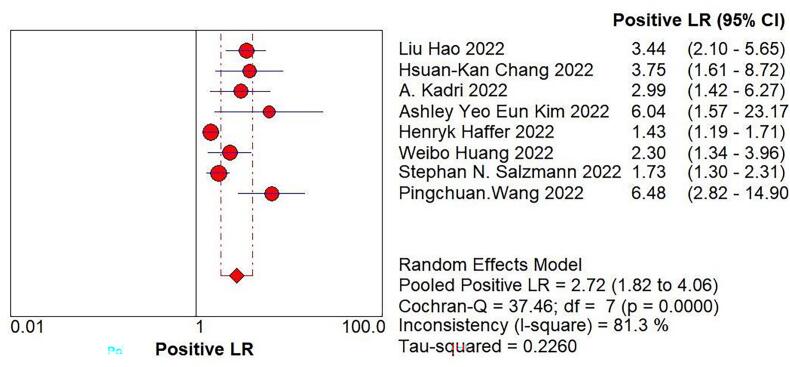

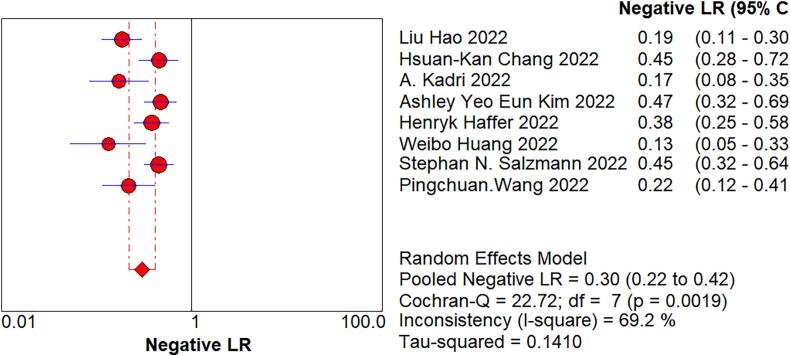

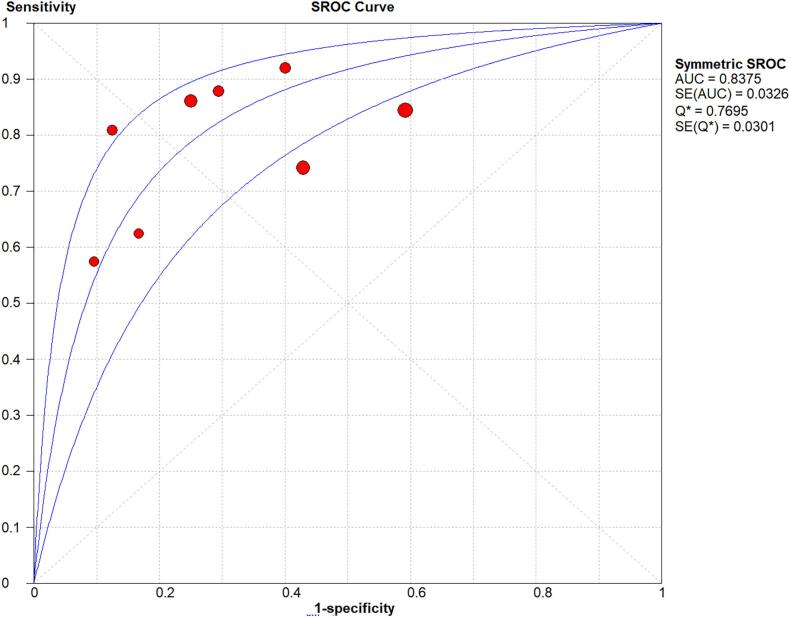

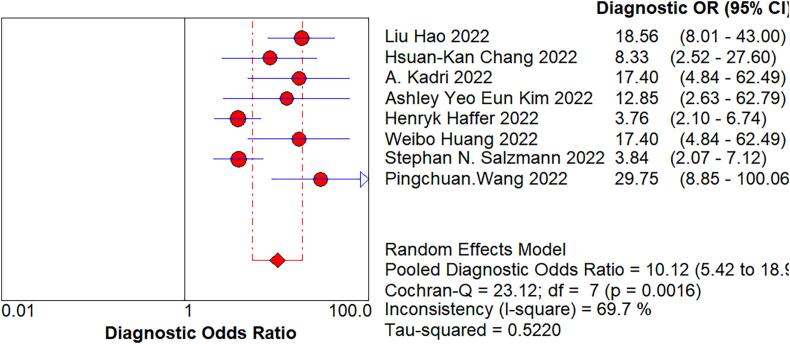
The pooled sensitivity, specificity, PLR, NLR, AUC, and DOR.

We imported the current data into Meta DiSc software, and yielded a Spearman correlation coefficient of 0.500 between the log of sensitivity and the log of (1-specificity), (p=0.207 > 0.05), which was not significant, implying that there was no threshold effect in this study. Further, by plotting the symmetric sROC curve, there was no “shoulder-arm shape”, which further illustrated the absence of a threshold effect in this study. The Cochran-Q test for the DOR yielded Cochran-Q = 23.12, p > 0.001, implying that there was no heterogeneity due to non-threshold effects in this study. Further, the $I^{2}$ of sensitivity, specificity, PLR, NLR, and DOR in this study were all >50%, so the random effects model was used for the combination of the above five evaluation indicators. The pooled sensitivity was 0.809 (95% CI, 0.777–0.838, $I^{2}$ = 78.8%), the pooled specificity was 0.640 (95% CI, 0.587–0.691, $I^{2}$ = 85.9%), the pooled PLR was 2.721 (95% CI, 1.825–4.056, $I^{2}$ = 81.3%), the pooled NLR was 0.301 (95% CI, 0.218–0.415, $I^{2}$ = 69.2%), the pooled AUC was 0.8375, and the Q-value = 0.7695; the DOR was 10.119 (95% CI, 5.419–18.896, $I^{2}$ = 69.7%) (Fig. 4).

## 4 Discussion

Osteoporosis is a very common disease characterized by low bone mass and destruction of bone microarchitecture and it can lead to fragility fractures (Rachner et al., 2011). With an aging population, osteoporosis is increasingly becoming a global epidemic. It is a serious public health problem (Sözen et al., 2017). Because most people have no obvious symptoms of osteoporosis before a fracture, many people are not diagnosed in time to receive effective prevention and treatment in the early stages. An International Osteoporosis Foundation (IOF) survey, one of the main reasons for the under-diagnosis and under-treatment of patients with osteoporosis is the limited opportunity for diagnosis before the first fracture (Lewiecki et al., 2019). Therefore, an early and accurate diagnosis of osteoporosis is extremely crucial. The diagnosis of osteoporosis is currently determined by measuring BMD by DXA of the proximal femur and lumbar spine, or in the absence of significant trauma, by fracture of the femur or spine in adults (Di Paola et al., 2019). However, some factors such as obesity, vertebral disease (osteophytes, scoliosis, or fractures), or external artifacts (aortic calcification or surgical internal fixation) may affect the BMD results during measurement, reducing the accuracy of the results and causing a certain percentage of missed diagnoses (Adams, 2013). Meanwhile, a study found that only 10%–53% of BMD measured in postmenopausal women over 65 years of age after a fragility fracture met the osteoporosis diagnostic criteria for osteoporosis (McNamara, 2010).

With the rapid development of imaging technology, many imaging methods have emerged in the diagnosis and assessment of osteoporosis, such as quantitative CT (Engelke et al., 2008), peripheral quantitative CT (Sheu et al., 2011), digital x-ray radiogrammetry (Kälvesten et al., 2016) and quantitative US (Nayak et al., 2006), etc. Although DXA T-scores has been commonly used as a criterion for defining osteoporosis, it is a two-dimensional (2D) measure that only measures density/area (in grams per square centimeter) and areal bone density which is susceptible to bone size, thus overestimating fracture risk in individuals of short stature. DXA is also sensitive to degenerative changes, and individuals with the substantial degenerative disease have increased areal density, resulting in a lower predicted fracture risk than exists; and all structures on the spine can affect bone density, such as aortic calcification, morphological abnormalities, and after spinal laminectomy, etc. (Link, 2012).

Studies have shown that the occurrence of osteoporosis is often accompanied by an increase in bone marrow adipocytes and changes in the content of a variety of bone marrow adipose tissues (Justesen et al., 2001). Therefore, changes in adipocytes within the bone marrow can indirectly reflect bone quality, and the use of MRI techniques can provide an early, comprehensive, and noninvasive assessment of bone marrow fat content and changes (Chang et al., 2017). With the onset of osteoporosis, the signal in the portion of the bone trabeculae in the T1-weighted image of MRI becomes stronger, which due to the increased signal when fat infiltrates into the bone, as early study confirmed that osteoporosis was characterized by atrophy of the bone trabeculae and localized replacement of the adipocytes (Bloem et al., 2018). The degree of fat infiltration in the vertebral body of patients with osteoporosis is highly correlated with the signal intensity of T1-weighted images (Shah and Hanrahan, 2011b), and the more severe the fat infiltration inside the vertebral body, the more severe the degree of osteoporosis and impaired bone quality. The elevated fat content produces a higher SI on T1w MRI due to fat having a shorter relaxation time compared to water, producing a bright and dark signal, respectively. So the degree of osteoporosis can be assessed by using MRI T1-weighted images (Chang et al., 2017). Based on this, Bandirali et al. introduced an MRI-based score for diagnosing osteoporosis, named the M-score, which has an accuracy of 84.4% in discriminating osteoporosis from non-osteoporosis (Bandirali et al., 2015). However, the M-score was limited in its clinical application because it was specific to a single MR platform, and M-score scores vary between devices. To eliminate this influence, Ehresman et al. (2020a) further investigated the T1-weighted image and proposed the VBQ score. VBQ score is a non-invasive, non-ionizing imaging modality, it is simple and fast. It is normalized to the patient by using the CSF, which is similar in composition across individuals (Spector et al., 2015; Damkier et al., 2010), so it can be used on different types of MR scanners. More recently, the method has been applied in many aspects in lumbar spine surgery and proved its satisfying efficacy on prediction of fragility fractures in at-risk patients, indicating bone quality in patients with degenerative diseases or vertebral compressive fractures (Ehresman et al., 2020a; Ehresman et al., 2021; Salzmann et al., 2022; Ehresman et al., 2020b). Ehresman et al. conducted a retrospective cohort study of patients undergoing spine surgery for degenerative disease to evaluate the correlation between the VBQ score and DXA T-score, and the correlation between the VBQ score and osteopenia/osteoporosis. It was concluded that the higher the VBQ score, the higher the prevalence of osteopenia/osteoporosis, and this score was a significant predictor of healthy bone versus osteopenia/osteoporosis with an accuracy of 81% (Ehresman et al., 2020a). Ehresman et al. showed that an arbitrary cut-off value of VBQ >3 was useful in recognizing patients at-risk for revision surgery (Ehresman et al., 2020b). The thresholds for the VBQ scores for the studies we included ranged from 2.39 to 3.08. The study by Kadri et al. revealed that VBQ >3.0 can be used to identify patients who need further diagnostic evaluation (Kadri et al., 2022). Wang et al.'s study showed that the cut-off value of diagnose abnormal bone mass was 2.98, with a sensitivity of 81.6% and a specificity of 88.6% (王平川, 王俊武, 石鹏志, 等, 2022). Kim et al. concluded that the cut-off VBQ score for osteoporosis was calculated to be 2.6 (58% sensitivity, 90% specificity) with a Youden Index of 0.484 (Kim et al., 2023).

The purpose of our study was to explore the value of MRI-based VBQ scores for evaluating osteoporosis, so we included a total of 999 patients in 8 original trials in this study, and the VBQ score had a high accuracy of 0.8375 for the diagnosis of bone loss, with a sensitivity of 0.809 and a specificity of 0.640. Thus, the VBQ score can be used as a new tool for screening for bone loss. Some previous studies were similar to our conclusion. Salzmann et al. concluded that using the VBQ score to differentiate between patients with osteopenia/ osteoporosis BMD and normal BMD had a cut-off value of 2.388, yielded a sensitivity of 74.3% and specificity of 57.0% with an AUC of 0.7079, it showed moderate diagnostic power (Salzmann et al., 2022). Haffer et al. analyzed 267 patients and the ROC analysis for the identification of osteoporosis/ osteopenia had a sensitivity of 84.7% and specificity of 40.6% at a VBQ score threshold of 2.18. They suggested that the VBQ score may be a new non-invasive tool for evaluating bone quality (Haffer et al., 2022). Kim et al. conducted a retrospective study of patients who performed low back surgery between 2016 and 2021 (n =61). The ROC analysis revealed that higher VBQ scores were associated with the presence of osteoporosis (AUC =0.754, p =0.006). The cut-off of VBQ score for osteoporosis was 2.6 (Youden index 0.484; sensitivity: 58%; specificity: 90%). They found that the VBQ score was a significant predictor of osteoporosis and could distinguish between healthy and osteoporotic vertebrae (Kim et al., 2023). Some scholars believe that VBQ score can be used as an opportunistic tool to assess bone quality before back surgery. Kadri et al.'s retrospective study evaluated 83 patients aged ≥50 years scheduled for thoracolumbar spine surgery who received T1-weighted MRI. They identified a VBQ score of 2.95 as a diagnostic threshold with optimal sensitivity and specificity to detect osteoporosis based on DXA T-scores. They concluded that MRI is a simple, effective tool that may help identify patients at risk of developing complications related to bone disease, and that patients need additional diagnostic evaluation when VBQ > 3.0 (Kadri et al., 2022). Pu et al.'s study included 100 patients with preoperative lumbar disc herniation. Their study found an optimal diagnostic threshold for bone loss with VBQ scores of 3.06 (sensitivity 0.636, specificity =0.870, positive predictive value [PPV] =0.942, negative predictive value [NPV] =0.417) and 3.05 (sensitivity =0.875, specificity =0.618, PPV =0.519, NPV =0.913). The VBQ score provided an additional screening opportunity for preoperative BMD assessment, with a VBQ score <3.05 essentially ruling out osteoporosis, while a VBQ score ≥3.05 (especially ≥3.06) indicated the need for further investigations (Pu et al., 2023). Some believed that the VBQ score also be used as a predictor of fracture in patients with osteoporosis, Ehresman et al. demonstrated that MRI-based VBQ score was both an independent predictor of vulnerability fracture in high-risk patients and a superior predictor of fracture risk compared to BMD as measured by DXA (Ehresman et al., 2021). Li et al. collected 196 patients and assigned them to osteoporotic vertebral compression fracture (OVCF) and non-OVCF groups, and compared them between age groups. It was concluded that the VBQ score was a valid measure of bone quality in OVCF patients, comparable to the T-score, especially in those over 60 years old (Li et al., 2022). Stephan et al. included a total of 156 patients with hyperlipidemia and DXA showed elevated baseline VBQ score in patients with healthy bone density (p <0.001) and the AUC for predicting VBQ score for osteoporosis was more consistent with the DXA results after controlling for hyperlipidemia (AUC =0.72, 0.70 vs. AUC =0.88, 0.89; p <0.001). Their concluded that hyperlipidemia increased VBQ score in healthy skeletal population. This suggested that physiological variables affecting bone composition may influence VBQ score (Aynaszyan et al., 2022). Therefore, we should investigate the effect of hyperlipidemia on the diagnosis of VBQ score for reduced bone mass. From a practical standpoint, the novel VBQ score has several potential advantages. First, the VBQ measurement is relatively fast and simple to perform. It can be performed with standard PACS software without any additional commercial software. In addition, because it is based on MRI data that most spine surgery patients routinely receive prior to surgery, it could improve clinical screening rates at no additional cost or radiation exposure. Lastly, VBQ score might be a more sensitive risk assessment tool compared to measurements of bone “quantity” using BMD. As shown in the Rotterdam Study, less than half of the fractures in the elderly occurred in individuals with a DXA-based BMD classified as osteoporotic, with the majority of fractures occurring in individuals with a BMD classified as non-osteoporotic (Schuit et al., 2004).

Our study was the first to analyze the accuracy of MRI for the evaluation of reduced bone mass. Due to the low invasiveness of MRI, it had received a great deal of attention. However, this study had some limitations that should be carefully considered. Firstly, the total number of patients included in this study was 999, which was not a large enough sample size, so we need to include more samples to ensure the accuracy of the study findings. Secondly, most of the included patients were examined before back surgery, which led to the selection bias, but retrospective studies are susceptible to selection bias (which may lead to an overestimated diagnostic advantage ratio). Thirdly, the elliptical tool to place the ROI region has limit on its shape and direction so that more cancellous bone could be included by using more powerful ROI tools. Fourthly, current MRI scans were generally done in supine position so that the results might not entirely reflect the states in other positions. Fifthly, VBQ score only distinguished between normal and abnormal bone mass, and the ability to distinguish between osteopenia and osteoporosis does not yet meet clinical needs, and further research and improvement are needed. Sixthly, there was heterogeneity among these studies, with sensitivity and specificity being compromised. However, this was not uncommon in meta-analyses of diagnostic studies, as there were differences in thresholds for both clinical significance and reference standards. This study did not discuss the influence of population characteristics (such as race, sex, age, BMI, etc.) due to the limitations of the chosen studies. Due to the retrospective nature of this study bone specimens for histological analysis could not be obtained. Future studies including histological data might benefit our understanding of the biological nature of the VBQ score. It was also unknown if the results could be applied to the T-score of the femoral neck, or any other bone structures besides the lumbar spine. Further researches and information on different populations and anatomical locations are needed to confirm and generalize our pilot findings. Therefore, further prospective studies are necessary to explore the value of MRI-based VBQ scores for evaluating osteoporosis.

## 5 Conclusion

Overall, this meta-analysis suggested that MRI-based VBQ scores provided high sensitivity and moderate specificity in detecting osteoporosis. Opportunistic use of VBQ scores could be considered, e.g. before lumbar spine surgery.

## CRediT authorship contribution statement

**Chen Ang:** Writing – review & editing, Writing – original draft, Software, Resources, Methodology, Investigation, Formal analysis, Data curation, Conceptualization. **Feng Shangyong:** Validation, Investigation, Formal analysis, Data curation. **Lai Lijuan:** Resources, Investigation, Formal analysis, Data curation. **Yan Caifeng:** Writing – review & editing, Supervision, Resources, Project administration, Funding acquisition, Data curation.

## Declaration of competing interest

No potential conflict of interest was reported by the authors.

## Data Availability

The data that has been used is confidential.
